# Effects of sweating on distal skin temperature prediction during walking

**DOI:** 10.1186/2046-7648-4-S1-A31

**Published:** 2015-09-14

**Authors:** Stephanie Veselá, Boris RM Kingma, Arjan JH Frijns

**Affiliations:** 1 Eindhoven University of Technology, Eindhoven, The Netherlands; 2 NUTRIM School, Maastricht University Medical Center, The Netherlands

## Introduction

Thermal sensation models require a high quality prediction of local skin temperatures (T_skin,X_) from thermoregulation models. However, most thermoregulation models are validated for T_skin,mean _under laboratory setting. The objective of this study is to investigate the challenges of simulating distal skin temperatures T_skin,distal _during walking.

## Methods

For this study, the skin temperature (T_skin_) of human subjects (4 males, 2 females) is measured at 15 sites (locations according to [1] plus fingertip) while walking indoors (2.8 met). The subjects wear an everyday outfit consisting of underwear, jeans, T-shirt, long-sleeved shirt, socks and shoes (0.8 clo) [2]. The temperature is recorded every 60 seconds during a one hour experiment. The measured data is then compared to the computed T_skin,X _of the mathematical thermoregulation model ThermoSEM [3].

## Results

**Figure 1:**
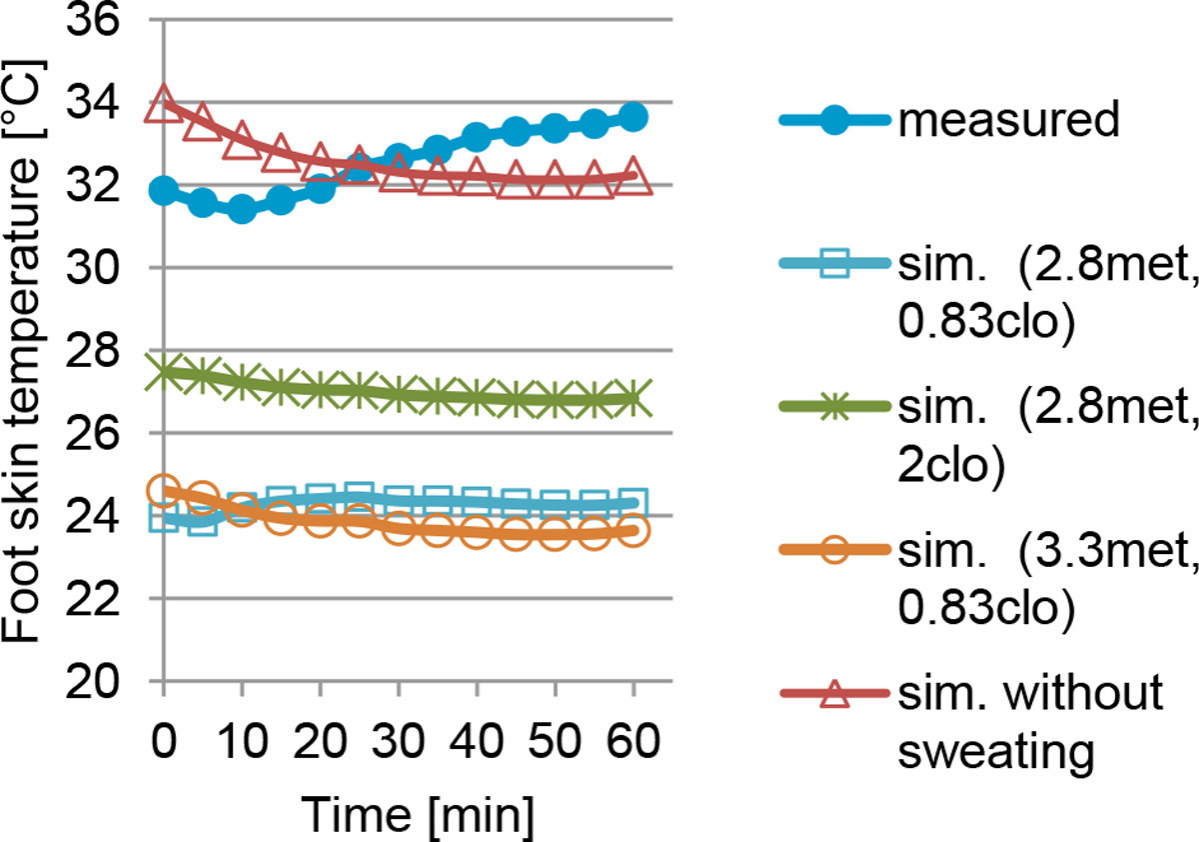
**Measured and simulated foot temperature (Tskin, foot) for one male subject while walking indoors (5 minute average)**. The effects of increased clothing insulation and metabolic rate as well as neglecting sweating are shown.

The computed T_skin,mean _are within 2 °C of the measured temperatures. The measured T_skin,foot _range from 29 °C to 34 °C for all subjects with an increase of 2-3 °C in the course of one hour walking. The computed T_skin,foot _largely underestimate the measured values by 4 to 9 °C (Figure 1, light blue squares). For T_skin,hand _it differs only 1 to 4 °C. The clothing insulation and metabolic activity are estimates and might differ from reality. By raising the clothing insulation at the foot to a maximal measured value of 2 clo (see [2]) the computed temperatures increase by 3 °C (Figure 1, green crosses). The increase of metabolic rate leads to slightly lower computed T_skin,foot _(Figure 1, orange circles). Lower T_skin,foot _at increased metabolic rate is due to evaporative heat losses over the entire body because of sweating. If the sweating is neglected in the model, the computed and measured results are in better agreement (Figure 1, red triangles).

## Discussion

Even though the exclusion of sweating leads to improved results for T_skin,foot_, the main issue is the latent heat transport from the foot skin surface to the environment. The current clothing model only includes a total evaporative resistance taken from [4] due to the absence of studies on detailed local evaporative resistances. Therefore new experiments on local (evaporative) clothing resistances are needed.

## Conclusions

In order to account for the reduced heat losses when wearing vapour resistant clothing (e.g. shoes), clothing models should differ between sensible and latent heat transport from the skin to the clothing and from the clothing to the environment. Furthermore, experiments are required to quantify the local evaporative resistances more accurately.

## References

[B1] ISO EN-ISO 9886. Ergonomics - evaluation of thermal strain by physiological measurements 2004

[B2] Lee J Zhang H Arens E Typical Clothing Ensemble Insulation Levels for Sixteen Body Parts In CLIMA Conference 2013 9

[B3] Kingma BRM Incorporating neurophysiological concepts in mathematical thermoregulation models Int J Biometeorol 2014 58 87 99 10.1007/s00484-012-0628-5 23354424

[B4] ISO EN-ISO 9920. Ergonomics of the thermal environment - Estimation of the thermal insulation and water vapour resistance of a clothing ensemble 2007

